# Insulinoma Unmasked Post Sleeve Gastrectomy With Incidental Renal Cell Carcinoma: A Rare Case

**DOI:** 10.7759/cureus.60395

**Published:** 2024-05-15

**Authors:** Yashika Goel, Utkrant Kurlekar, Ashish Chitharanjan, Amruta N Beke

**Affiliations:** 1 Department of General Surgery, Deenanath Mangeshkar Hospital and Research Center, Pune, IND; 2 Department of General Surgery, Bariatric Surgery, and Surgical Oncology, Deenanath Mangeshkar Hospital and Research Center, Pune, IND; 3 Department of Surgical Oncology, Deenanath Mangeshkar Hospital and Research Center, Pune, IND

**Keywords:** renal cell carcinoma (rcc), post bariatric surgery hypoglycemia, von hippel-lindau syndrome (vhl), pancreatic neuroendocrine tumor (pnet), insulinoma

## Abstract

Insulinoma is a functional pancreatic neuroendocrine tumor (pNET). Usually benign and solitary, these tumors present with recurrent episodes of hypoglycemia due to insulin hypersecretion. It’s a rare cause of post bariatric surgery hypoglycemia and hence poses a diagnostic challenge.

Here, we report the first case of a 53-year-old male with insulinoma unmasked post sleeve gastrectomy with incidental renal cell carcinoma (RCC). He presented with symptoms of Whipple’s triad after two months of sleeve gastrectomy done for morbid obesity. On further inquiry, the patient gave a history of an asymptomatic peripancreatic neuroendocrine tumor (NET) for the past 11 years. With a suspicion of insulinoma, biochemical workup followed by non-invasive imaging like GA-68 DOTA and CT triphasic abdomen scan was done to guide the diagnosis of an insulinoma which also detected a second primary tumor in the right kidney, likely to be a malignant RCC. Following pancreatic mass excision with radical nephrectomy for right renal mass, histopathology (HPE) and immunohistochemistry (IHC) confirmed the diagnosis of an insulinoma and a right renal clear cell carcinoma. The patient was discharged with no further episodes of hypoglycemia. Hence, persistent hypoglycemia post bariatric surgery could be an indication of a hidden insulinoma and this possibility of synchronous tumors should be kept in mind when dealing with rare tumors like insulinoma.

## Introduction

Insulinoma is a rare type of functional pancreatic neuroendocrine tumor (pNET) characterized by Whipple’s triad - symptomatic hypoglycemia, low plasma glucose concentration at the time of symptoms, and relief of symptoms with administration of glucose. It is one of the rare causes of fasting hypoglycemia post bariatric surgery. A literature search showed 21 patients diagnosed with insulinoma post bariatric surgery, out of which eight patients (all female) had presented after sleeve gastrectomy [1]. These functional pNET’s can present with synchronous tumors which are either sporadic or seen as a part of hereditary cancer syndromes like Von Hippel-Lindau (VHL). One such case of sporadic occurrence of insulinoma with renal cell carcinoma (RCC) has been reported so far [2].

Here, we report the first case in a male patient with insulinoma as a cause of persistent fasting hypoglycemia unmasked after bariatric surgery (sleeve gastrectomy) with incidentally detected RCC at the same time.

## Case presentation

A 53 year-old-male operated on for sleeve gastrectomy outside of our institute in October 2022, came with complaints of recurrent episodes of hypoglycemia characterized by dizziness, fatigue, disorientation, and falling episodes. He lost 20 kg weight after surgery and started to have these symptoms after two months of bariatric surgery in December 2022. These episodes occurred during periods of prolonged fasting and there was immediate improvement of symptoms after oral intake of glucose. Prior to sleeve gastrectomy, his laboratory tests showed blood sugar level (BSL) levels of 55 mg/dL and serum C-peptide level of 11.7 ng/mL (elevated ). After sleeve gastrotomy, laboratory tests done in March 2023 showed normal levels of serum beta-hydroxybutyrate (0.24 mmol/L), serum fasting insulin levels (4.61 µU/mL), serum fasting C-peptide (1.67 ng/mL). On further inquiry, he gave a history of a long-standing peripancreatic mass of size 2.5x2 cm incidentally detected on a routine USG abdomen 11 years ago. Endoscopic ultrasonography (EUS) and fine needle aspiration (FNA) done at that time were suggestive of a low-grade neuroendocrine tumor (NET) grade 1. Since the patient was asymptomatic and the size was small, he was advised observation for the same. Now, with suspicion of an insulinoma, the patient was subjected to a 72-h fasting test. After 8 h of fasting, he developed symptomatic hypoglycemia with random BSL 22 mg/dL. Gallium 68 exendin scan done in April 2023 showed a soft tissue lesion measuring 34x45 mm in the gastrohepatic ligament (standardized uptake value {SUV} 37.8), abutting the neck of pancreas with increased GLP-1 expression suggestive of an insulinoma and a right renal cyst in the anterior cortex of size 62x48 mm. He was started on medical treatment, and a trial of octreotide injections was given but there was no symptomatic benefit. Then, he was referred to our center with the above-mentioned reports and was admitted for further management.

**Figure 1:**
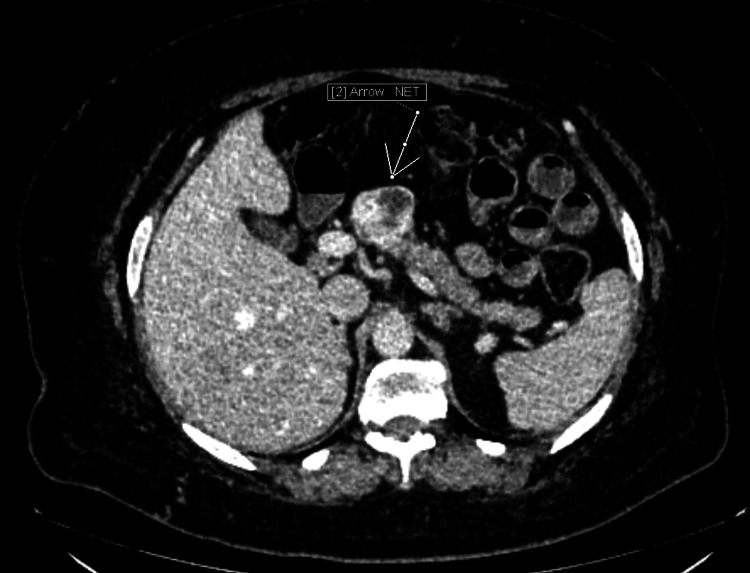
NET arising from the neck and proximal body of pancreas. NET: neuroendocrine tumor

**Figure 2:**
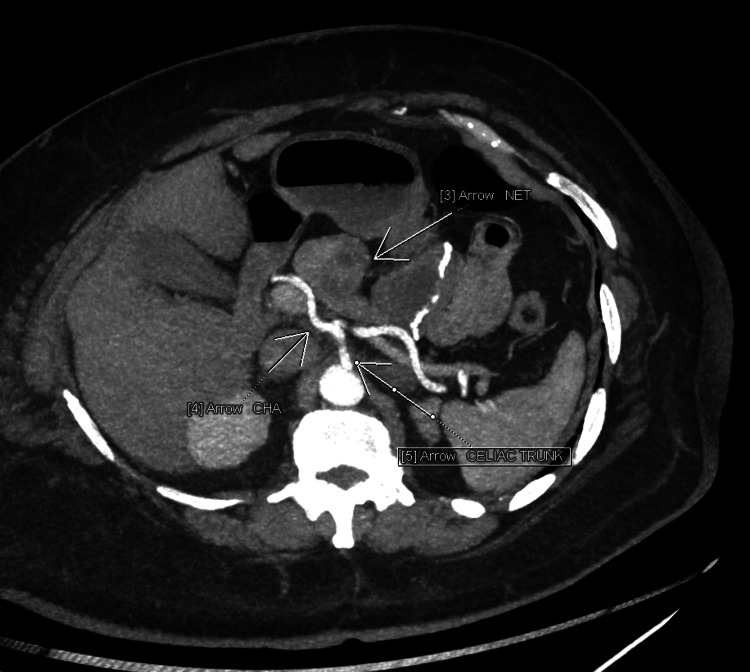
Completely exophytic pancreatic mass with maintained fat planes with CHA. CHA: common hepatic artery

**Figure 3:**
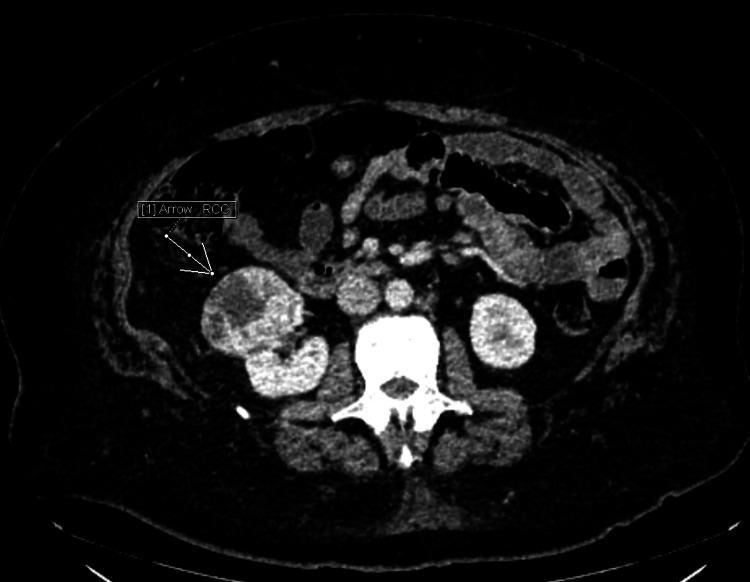
Mass arising from right kidney likely to be malignant RCC. RCC: renal cell carcinoma

Physical examination showed an obese male with a BMI of 41.49 kg/m^2^ (before sleeve gastrotomy his BMI was 63.7 kg/m^2^) and random BSL levels ranging from 39 mg/dL to 60 mg/dL. For preoperative optimization, he was started on corn starch powder orally 3 times a day and IV dextrose 10% with 2 hourly BSL monitoring. A triphasic CT scan of the abdomen showed a well-defined, heterogenous exophytic lesion arising from the neck-proximal body of the pancreas, 3.9x4.7x3.4 cm in size protruding into the lesser sac consistent with NET (Figure 1). Fat planes were maintained with the gastroduodenal and splenic arteries (Figure 2) as well as with splenic, portal, and superior mesenteric veins. The scan also showed a heterogeneous exophytic predominantly solid lesion in the anterior and inter-polar regions of the right kidney, measuring 5.5x5.0x4.6 cm in size located within 1 mm of renal collecting system without any enlargement of hilar nodes (Figure 3). The renal nephrometry score of the lesion was 8, likely to be of malignant etiology.

**Figure 4:**
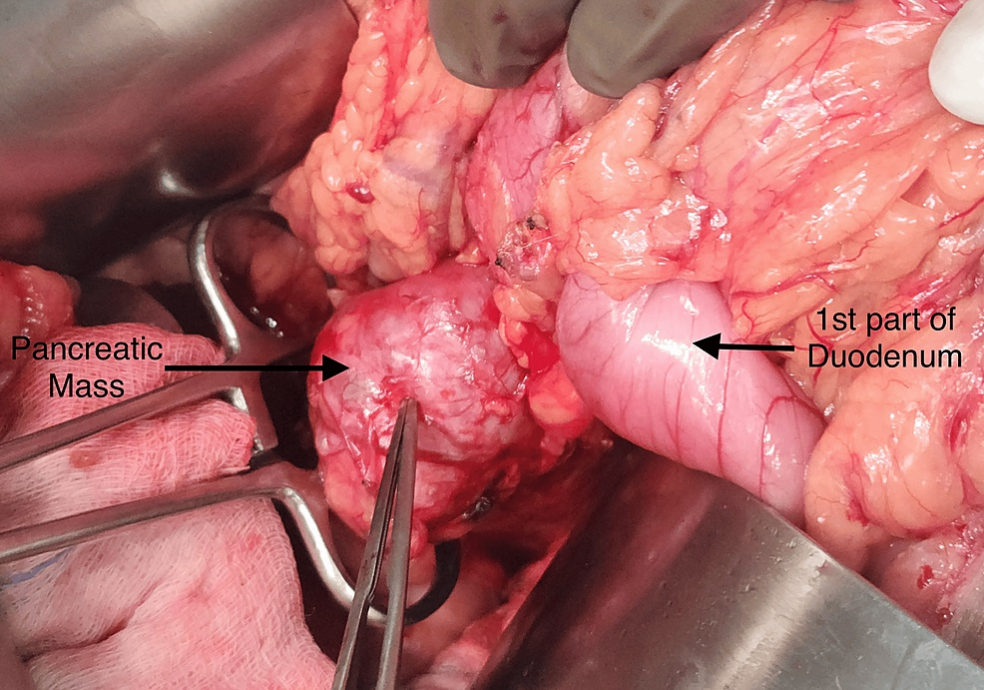
Pancreatic mass is seen just adjacent to the first part of duodenum after mobilization.

**Figure 5:**
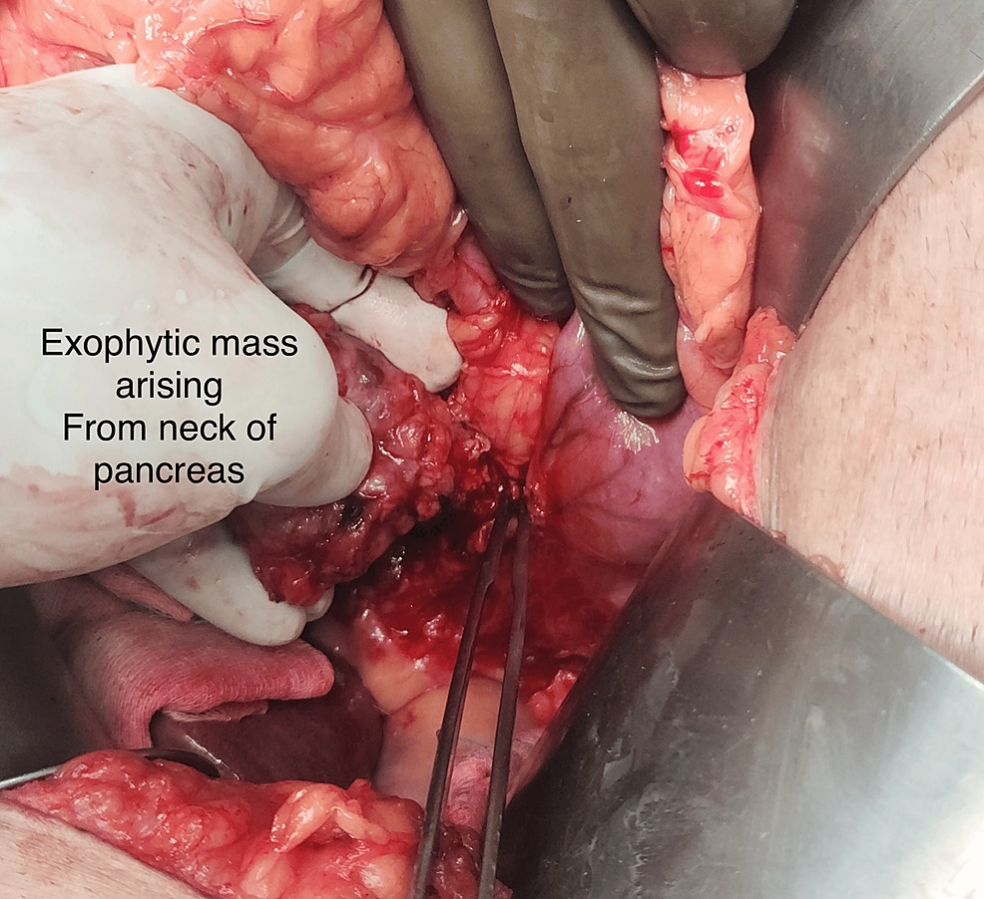
After releasing all the adhesions of the mass from duodenum, it was completely exophytic arising from the neck-proximal body of pancreas.

**Figure 6:**
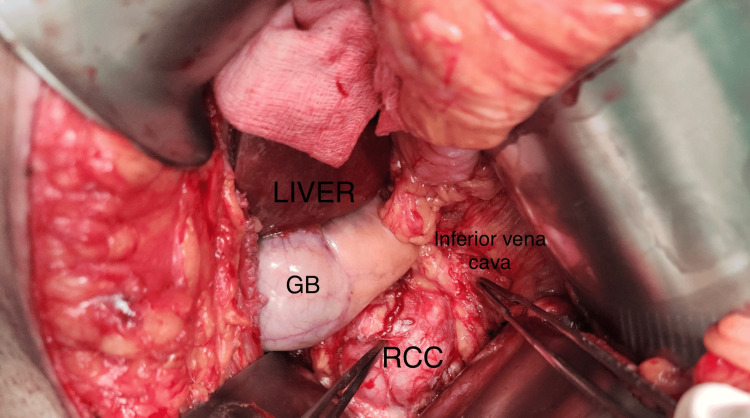
Right kidney mass was approached after pancreatic mass excision.

Family history pertaining to familial cancer syndromes like Von Hippel-Lindau (VHL) was negative. However, the patient did not consent to further workup. He was counseled regarding the two primary pathologies and was advised of surgery. He subsequently underwent an exploratory laparotomy with pancreatic mass excision (Figures 4, 5) and radical nephrectomy for the right renal tumor (Figure 6).

**Figure 7:**
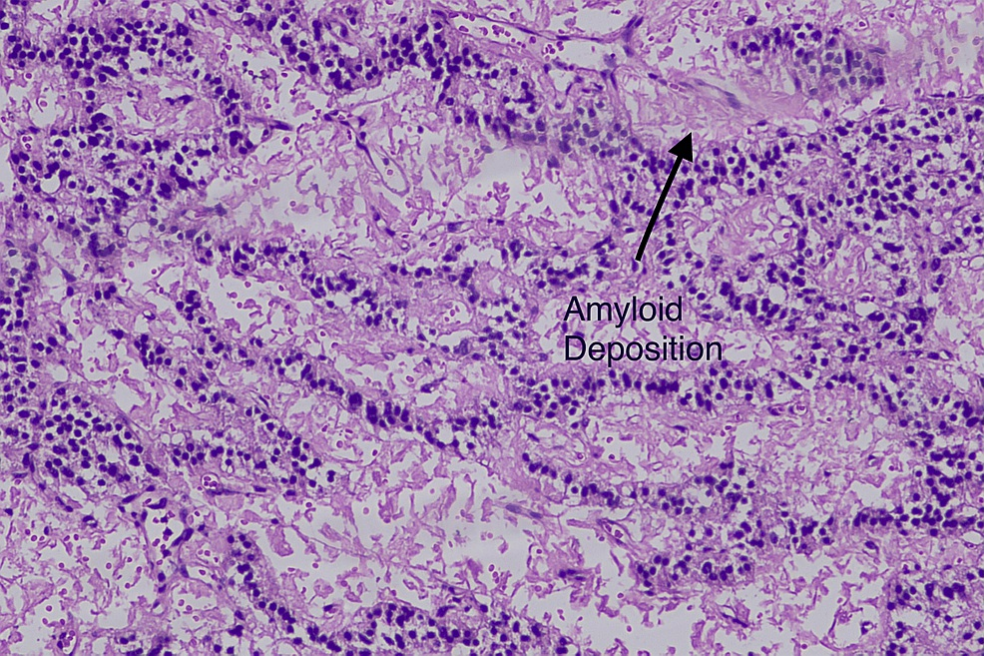
Pancreatic mass histopathology at a magnification of 10x. Monotonous cells arranged in trabecular, rosettes, and in cords with large areas of acellular eosinophilic amorphous material suggestive of colloid in intervening stroma, round to oval nuclei, stippled chromatin, moderate amount of amphophilic cytoplasm with occasional mitosis. No necrosis was noted, suggestive of a well-differentiated grade 1 neuroendocrine tumor.

**Figure 8:**
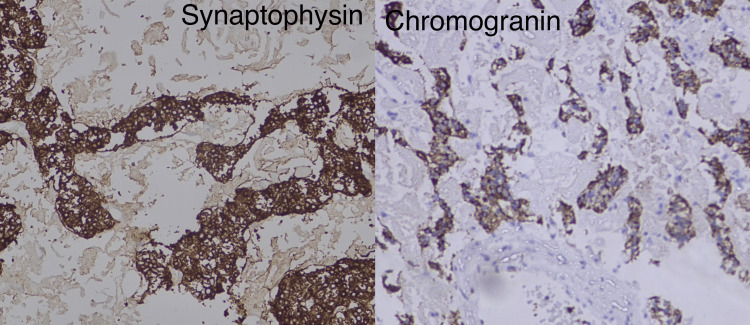
Cells showing strong positivity for synaptophysin and chromogranin.

**Figure 9:**
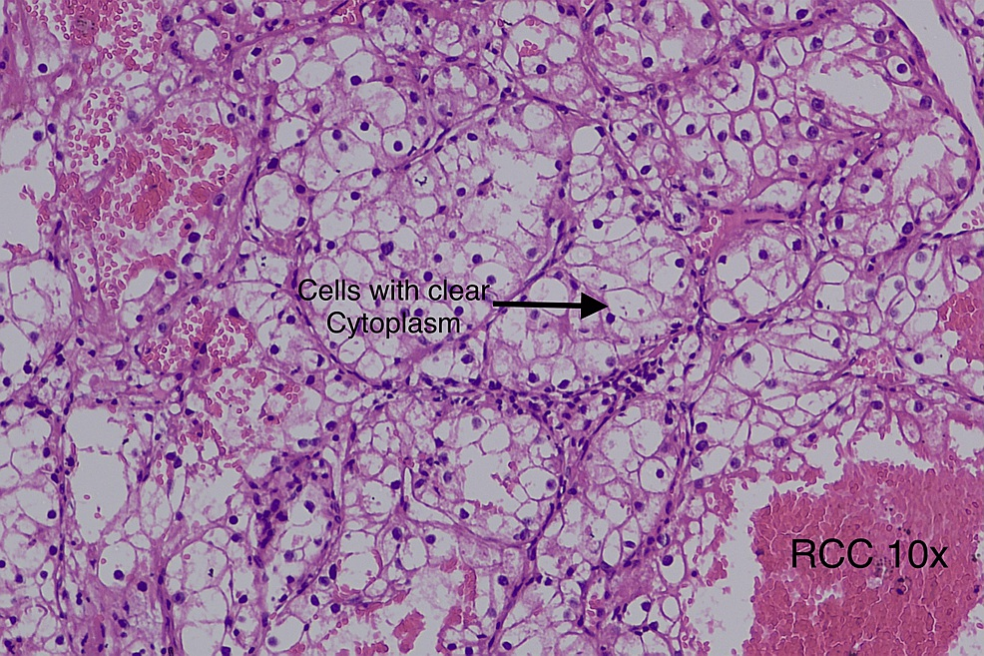
Renal tumor histopathology at a magnification of 10x. Clear cells with areas of eosinophilic change, arranged in sheets and papillary pattern interspersed with fine vascular network, 20-30% necrosis, areas of hemorrhage, cystic degeneration, focal calcification suggestive of clear cell renal carcinoma.

Histopathology and immunohistochemistry confirmed the diagnosis of a well-differentiated low-grade neuroendocrine tumor of the pancreas of size 5x4.3x3 cm with intact capsule limited to the pancreas with mitotic count less than 1/hpf (WHO G1, pT3Nx) (Figure 7), mass showed strong positivity for chromogranin, synaptophysin, and ATRX (Figure 8). The kidney tumor histopathology showed clear cell carcinoma of right kidney of size 5.2x4.5x3.5 cm, unifocal, located at interpolar region, not invading the renal sinus fat nor pelvicalyceal system or Gerota’s fascia. Cut margins of the ureter and renal vein were free of tumors and all the excised lymph nodes were free of tumors (WHO/International Society of Urology Pathology {ISUP} nuclear grade 2, pT1bN0) (Figure 9). Immunohistochemistry (IHC) showed strong positivity for CK, vimentin, CD10, PAX8, and carbonic anhydrase.

Immediately after surgery, glucose levels increased to a normal range. There were no further symptoms of hypoglycemia and the patient was discharged in stable condition after seven days. On a three-month follow-up, his random BSL levels were recorded between 200 mg/dL and 250 mg/dL, and the patient has been started on oral hypoglycemic agents (OHA). The patient has been disease-free on a six-month follow-up after the surgery.

## Discussion

Hypoglycemia is defined as a fall in BSL below 70 mg/dL according to the American Diabetes Association guidelines [3]. Hypoglycemia is a complication after bariatric surgery, most commonly seen after Roux-en-Y gastric bypass (RYGB) surgery and rarely after sleeve gastrectomy (SG). The most common causes are late dumping syndrome, nesidioblastosis (both cause postprandial hyperinsulinemic hypoglycemia, which typically presents 1-3 h after food), and rarely, insulinoma (persistent prolonged fasting hypoglycemia) [4].

Insulinomas are the most common functional pancreatic neuroendocrine tumor (0.1-0.3 cases per 100,000 individuals per year) [5]. Most insulinomas are sporadic (usually detected around 50 years of age), solitary, benign, small <2 cm lesions (90%). However, 4-10% of cases are associated with hereditary cancer syndromes like MEN 1 which is more likely to be multifocal and present at an earlier age [6]. It presents with the features of Whipple's triad, namely hypoglycemic symptoms (neuroglycopenic or autonomic symptoms), low blood glucose level of less than 50 mg/dL, and relief of symptoms after glucose administration [7].

A 72-h fasting is a gold standard test for diagnosing insulinoma. Using the Endocrine Society Clinical Practice Guidelines (ESCPG), BSL equal to or below 55 mg/dL, insulin levels greater than or equal to 3 µU/mL, C-peptide level greater than or equal to 0.6 ng/mL, proinsulin level greater than or equal to 5 pmol/L, beta-hydroxybutyrate level less than or equal to 2.7 mmol/L during fasting, and absence of sulphonylurea, these factors help confirm the diagnosis of insulinoma [5,8]. A comprehensive study discussing the clinical implication of these criteria reported that by meeting two of the criteria out of four, mainly plasma glucose level and C-peptide level, the sensitivity and specificity could be 100 and 83%, respectively [9].

AlQambars et al. reported a case of insulinoma symptoms unmasked after biliopancreatic diversion (BPD). The likely explanation they advocated was that severe insulin resistance due to morbid obesity compensates for hyperinsulinemia before surgery and hence, patients are asymptomatic. Postoperative weight loss improves insulin sensitivity and patients start to experience progressive hypoglycemic symptoms [10].

Preoperative localization of tumors is important for optimal management in cases of insulinoma. It can be done by non-invasive modalities like abdominal USG, CECT, or MRI, or by invasive modalities like endoscopic ultrasonography (EUS) and intra-arterial calcium stimulation with hepatic venous sampling. CT scan is the first choice for localization with higher sensitivity compared to MRI, although MRI can be useful in the detection of small tumors that are not seen on CT. Due to the high density of GLP-1 receptors and its radiolabeled analogs like exendin 4 receptors on insulinoma, GLP-1 receptor scans like Ga 68 DOTANOC PET-CT, Ga 68 DOTATATE PET-CT, and Ga 68 NOTA-exendin-4 PET-CT are being used for localization of insulinoma with sensitivity upto 98%. EUS has a sensitivity of 94% alone and 100% when combined with CT. It also allows tissue diagnosis which can be very helpful, especially in small tumors [11].

Surgery is the treatment of choice for insulinoma. However, patients can be given a trial of somatostatin analogs and diazoxide preoperatively. The choice of surgery depends on the location of tumor. A completely exophytic lesion, or one that is not close to the main pancreatic duct, can be enucleated. While anatomical pancreatic resections are necessary in cases where the tumor is embedded deep in the pancreatic parenchyma or close to the pancreatic duct. Enucleation, when done, preserves the pancreatic parenchyma and prevents pancreatic insufficiency [12].

In this case, a CT scan of the abdomen, when done, also showed a right renal mass with malignant features. This raised the following two possibilities: pancreatic metastasis from RCC or a double primary tumor, either sporadic or related to hereditary cancer syndrome like VHL. Pancreatic metastasis is usually asymptomatic and multiple, occurs in the seventh decade of life following the greatest disease-free interval, mean time interval for detection is greater than 10 years, and a period as long as 32.7 years has been seen [13]. Also, in view of a prior history of low-grade NET, pancreatic metastasis was ruled out in view of low clinical suspicion.

VHL is an autosomal dominant inherited (80%) genetic condition due to an abnormal VHL gene characterized by hemangioblastomas of the brain, spinal cord, and retina; renal cysts and RCC; pheochromocytoma and paraganglioma; and pNETs with a positive family history. Usually, they develop symptoms starting from the second to third decade of life [14]. Pancreatic neuroendocrine tumor is seen in 9-17% of cases [15] and is usually non-functional and slow-growing, although, can be malignant, especially if the size is large [16]. Genetic testing is recommended in patients with more than one of the following tumors: RCC, pNET, serous pancreatic cystadenoma, and epididymal/adnexal cyst [14]. In view of the contrasting features of this patient compared to those mentioned above, the possibility of VHL was unlikely; however, the patient did not consent to VHL testing.

For stage 1 RCC, surgical resection is the curative and accepted treatment. In appropriately selected patients, partial nephrectomy has oncologic outcomes that are comparable with radical nephrectomy [17]. After insulinoma excision, patients can have elevated BSL due to suppression, atrophy, and degranulation of the remaining beta-pancreatic cells, effects of glucagon, glucocorticoids, and growth hormone in the immediate postoperative period. At a later date, pancreatic insufficiency, hidden DM, or unmasked other causes of insulin resistance can cause elevated BSL [18].

## Conclusions

Postoperative hypoglycemia is a known complication of bariatric surgery, particularly Roux-en-Y gastric bypass surgery. It occurs rarely after sleeve gastrectomy and is commonly associated with dumping syndrome. But persistent prolonged hypoglycemia is a rare occurrence and can be an indication of a hidden insulinoma. Its association with fasting and good response to oral glucose intake should raise the possibility of insulinoma.

The beneficial effects of bariatric surgery have an unexpected side effect of causing hypoglycemia secondary to improved insulin sensitivity. Also, the possibility of other primary tumors should be kept in mind when dealing with rare tumors like insulinoma. Hence, we would recommend that it is mandatory to make an effort and plan well before any surgical procedure in such cases.
